# 3,3′-Dibutanoyl-1,1′-(*o*-phenyl­ene)dithio­urea

**DOI:** 10.1107/S1600536810001789

**Published:** 2010-01-23

**Authors:** Aamer Saeed, Naeem Abbas, Hummera Rafique, Michael Bolte

**Affiliations:** a Department of Chemistry, Quaid-i-Azam University, Islamabad 45320, Pakistan; b Institut für Anorganische Chemie, J. W. Goethe-Universität Frankfurt, Max-von-Laue-Strasse 7, 60438 Frankfurt/Main, Germany

## Abstract

The mol­ecular conformation of the title compound, C_16_H_22_N_4_O_2_S_2_, is stabilized by two intramolecular N—H⋯O hydrogen bonds. The crystal packing shows N—H⋯O and N—H⋯S hydrogen bonds.

## Related literature

For details of the biological activity of bisthio­ureas, see: Berkessel *et al.* (2006 ▶); Moloto *et al.* (2004 ▶). For their applications, see: Atia *et al.* (2005 ▶); Hu *et al.* (2009 ▶); Phetsuksiri *et al.* (2003 ▶). For the synthesis of the title compound, see: Succaw *et al.* (2005 ▶).
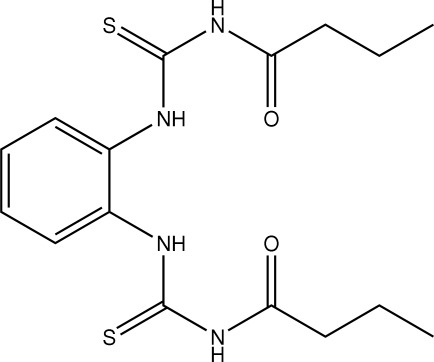


## Experimental

### 

#### Crystal data

C_16_H_22_N_4_O_2_S_2_
*M* *_r_* = 366.50
Monoclinic, 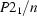

*a* = 8.8099 (5) Å
*b* = 16.4925 (7) Å
*c* = 12.3923 (8) Å
β = 91.949 (5)°
*V* = 1799.53 (17) Å^3^
*Z* = 4
Mo *K*α radiation
μ = 0.31 mm^−1^
*T* = 173 K
0.28 × 0.28 × 0.23 mm

#### Data collection

Stoe IPDS II two-circle diffractometer
Absorption correction: multi-scan (*MULABS*; Spek, 2009 ▶; Blessing, 1995 ▶) *T*_min_ = 0.918, *T*_max_ = 0.932
22483 measured reflections
3360 independent reflections
2890 reflections with *I* > 2σ(*I*)
*R*_int_ = 0.087

#### Refinement

*R*[*F* ^2^ > 2σ(*F* ^2^)] = 0.036
*wR*(*F* ^2^) = 0.095
*S* = 1.04
3360 reflections
229 parameters
H atoms treated by a mixture of independent and constrained refinement
Δρ_max_ = 0.23 e Å^−3^
Δρ_min_ = −0.33 e Å^−3^

### 

Data collection: *X-AREA* (Stoe & Cie, 2001 ▶); cell refinement: *X-AREA*; data reduction: *X-AREA* (Stoe & Cie, 2001 ▶); program(s) used to solve structure: *SHELXS97* (Sheldrick, 2008 ▶); program(s) used to refine structure: *SHELXL97* (Sheldrick, 2008 ▶); molecular graphics: *XP* in *SHELXTL-Plus* (Sheldrick, 2008 ▶); software used to prepare material for publication: *SHELXL97*.

## Supplementary Material

Crystal structure: contains datablocks global, I. DOI: 10.1107/S1600536810001789/bg2318sup1.cif

Structure factors: contains datablocks I. DOI: 10.1107/S1600536810001789/bg2318Isup2.hkl

Additional supplementary materials:  crystallographic information; 3D view; checkCIF report

## Figures and Tables

**Table 1:** Hydrogen-bond geometry (Å, °)

*D*—H⋯*A*	*D*—H	H⋯*A*	*D*⋯*A*	*D*—H⋯*A*
N11—H11⋯O1	0.86 (2)	1.90 (2)	2.6336 (17)	142.6 (17)
N12—H12⋯O2^i^	0.84 (2)	2.19 (2)	3.0309 (18)	175.3 (19)
N21—H21⋯O2	0.83 (2)	1.98 (2)	2.6616 (18)	139.1 (18)
N22—H22⋯S1^ii^	0.87 (2)	2.75 (2)	3.6147 (14)	172.0 (17)

Symmetry codes: (i) 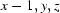; (ii) 
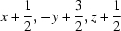
.

**Fig. 1.**
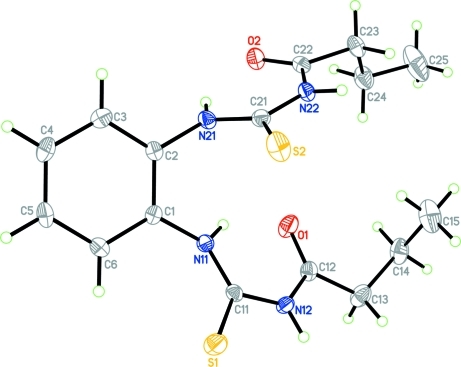
Molecular structure of title compound. Displacement ellipsoids are drawn at the 50% probability level.

## supplementary crystallographic information

### Comment

Various bisthiourea derivatives have attracted much attention due to their variety of applications and bioactivities. The presence of multivalent binding sites in bis thioureas provide a multitude of bonding possibilities. Urea and thiourea functionalities, presenting opportunities for the formation of diverse hydrogen bonded networks, represent powerful crystal engineering building blocks (Succaw *et al.*, 2005). The fluorinated bis-thiourea derivative are used as organocatalyst in Morita-Baylis-Hillman reaction (Berkessel *et al.*, 2006). *N*-alkyl thiourea Cadmium(II) complex as precursor for CdS-nanoparticle synthesis (Moloto *et al.*, 2004). BINOL (1,1'-Bi-2-naphthol) bis thiourea derivatives act as chemosensors (Hu *et al.*,2009). Bis-thiourea resins have been used for adsorption of silver(I) and gold(II) for application to retrieval of silver ions from processed photo films (Atia *et al.*, 2005). Diisoamyloxydiphenylthioureas are effective anti-tuberculosis agents (Phetsuksiri *et al.* (2003).

The molecular conformation of the title compound is stabilized by two N—H···O hydrogen bonds. The crystal packing shows N—H···O and N—H···S hydrogen bonds.

### Experimental

The compound was prepared acc ording to lierature procedure (Succaw *et al.*, 2005) and Recrystallized from methanol as colourless crystals: Anal. calcd.for C_16_H_22_N_4_O_2_S2: C, 52.43; H, 6.05; N, 15.29; S, 17.50%; found: C, 52.31; H, 6.19; N, 15.41; S, 17.62.

### Refinement

H atoms attached to C were geometrically positioned and refined using a riding model with C—H(aromatic) = 0.95 Å, *C*—*H*(methyl) = 0.98 Å, or *C*—*H*(methylene) = 0.99 Å, respectively. The position of the amino H atoms were freely refined. In all cases fixed individual displacement parameters

[U(H) = 1.2 *U*_eq_(C_aromatic_), 1.2 *U*_eq_(N); 1.5 *U*_eq_(C_methyl_)] were used.

### Crystal data

**Table:**

C_16_H_22_N_4_O_2_S_2_	*F*(000) = 776
*M**_r_* = 366.50	*D*_x_ = 1.353 Mg m^−^^3^
Monoclinic, *P*2_1_/*n*	Mo *K*α radiation, λ = 0.71073 Å
Hall symbol: -P 2yn	Cell parameters from 20465 reflections
*a* = 8.8099 (5) Å	θ = 3.4–26.0°
*b* = 16.4925 (7) Å	µ = 0.31 mm^−^^1^
*c* = 12.3923 (8) Å	*T* = 173 K
β = 91.949 (5)°	Block, colourless
*V* = 1799.53 (17) Å^3^	0.28 × 0.28 × 0.23 mm
*Z* = 4	

### Data collection

**Table:**

Stoe IPDS II two-circle diffractometer	3360 independent reflections
Radiation source: fine-focus sealed tube	2890 reflections with *I* > 2σ(*I*)
graphite	*R*_int_ = 0.087
ω scans	θ_max_ = 25.6°, θ_min_ = 3.4°
Absorption correction: multi-scan (*MULABS*; Spek, 2009; Blessing, 1995)	*h* = −10→10
*T*_min_ = 0.918, *T*_max_ = 0.932	*k* = −19→18
22483 measured reflections	*l* = −15→14

### Refinement

**Table:**

Refinement on *F*^2^	Primary atom site location: structure-invariant direct methods
Least-squares matrix: full	Secondary atom site location: difference Fourier map
*R*[*F*^2^ > 2σ(*F*^2^)] = 0.036	Hydrogen site location: inferred from neighbouring sites
*wR*(*F*^2^) = 0.095	H atoms treated by a mixture of independent and constrained refinement
*S* = 1.04	*w* = 1/[σ^2^(*F*_o_^2^) + (0.0595*P*)^2^ + 0.139*P*] where *P* = (*F*_o_^2^ + 2*F*_c_^2^)/3
3360 reflections	(Δ/σ)_max_ = 0.001
229 parameters	Δρ_max_ = 0.23 e Å^−^^3^
0 restraints	Δρ_min_ = −0.33 e Å^−^^3^

### Special details

**Table:**

Geometry. All e.s.d.'s (except the e.s.d. in the dihedral angle between two l.s. planes) are estimated using the full covariance matrix. The cell e.s.d.'s are taken into account individually in the estimation of e.s.d.'s in distances, angles and torsion angles; correlations between e.s.d.'s in cell parameters are only used when they are defined by crystal symmetry. An approximate (isotropic) treatment of cell e.s.d.'s is used for estimating e.s.d.'s involving l.s. planes.
Refinement. Refinement of *F*^2^ against ALL reflections. The weighted *R*-factor *wR* and goodness of fit *S* are based on *F*^2^, conventional *R*-factors *R* are based on *F*, with *F* set to zero for negative *F*^2^. The threshold expression of *F*^2^ > σ(*F*^2^) is used only for calculating *R*-factors(gt) *etc*. and is not relevant to the choice of reflections for refinement. *R*-factors based on *F*^2^ are statistically about twice as large as those based on *F*, and *R*- factors based on ALL data will be even larger.

### Fractional atomic coordinates and isotropic or equivalent isotropic displacement parameters (Å^2^)

**Table:**

	*x*	*y*	*z*	*U*_iso_*/*U*_eq_	
S1	0.07444 (5)	0.68569 (3)	0.35994 (3)	0.02446 (14)	
S2	0.47542 (5)	0.79179 (3)	0.52094 (3)	0.02947 (14)	
O1	0.38901 (14)	0.53284 (9)	0.57778 (9)	0.0278 (3)	
O2	0.83408 (13)	0.59749 (8)	0.58037 (9)	0.0224 (3)	
C1	0.42857 (18)	0.65251 (9)	0.31239 (12)	0.0152 (3)	
C2	0.57714 (18)	0.67920 (9)	0.33537 (12)	0.0154 (3)	
C3	0.66576 (19)	0.70843 (10)	0.25383 (13)	0.0206 (3)	
H3	0.7674	0.7249	0.2695	0.025*	
C4	0.6054 (2)	0.71355 (11)	0.14871 (13)	0.0228 (4)	
H4	0.6654	0.7342	0.0927	0.027*	
C5	0.4579 (2)	0.68847 (10)	0.12589 (12)	0.0211 (4)	
H5	0.4170	0.6924	0.0542	0.025*	
C6	0.36860 (19)	0.65746 (10)	0.20698 (12)	0.0180 (3)	
H6	0.2678	0.6399	0.1906	0.022*	
N11	0.34909 (15)	0.61572 (8)	0.39740 (10)	0.0160 (3)	
H11	0.401 (2)	0.5867 (12)	0.4430 (16)	0.019*	
C11	0.20588 (17)	0.62849 (10)	0.42462 (12)	0.0159 (3)	
N12	0.16371 (16)	0.58921 (8)	0.51872 (10)	0.0168 (3)	
H12	0.072 (2)	0.5945 (12)	0.5357 (15)	0.020*	
C12	0.25388 (19)	0.54519 (10)	0.59085 (12)	0.0195 (3)	
C13	0.1723 (2)	0.51260 (12)	0.68687 (14)	0.0272 (4)	
H13A	0.1316	0.4581	0.6691	0.033*	
H13B	0.0852	0.5484	0.7017	0.033*	
C14	0.2751 (2)	0.50692 (12)	0.78795 (14)	0.0285 (4)	
H14A	0.2206	0.4776	0.8446	0.034*	
H14B	0.3663	0.4748	0.7711	0.034*	
C15	0.3251 (3)	0.58888 (14)	0.83166 (17)	0.0447 (5)	
H15A	0.3912	0.5812	0.8961	0.054*	
H15B	0.2357	0.6205	0.8506	0.054*	
H15C	0.3809	0.6179	0.7765	0.054*	
N21	0.64067 (16)	0.67101 (9)	0.44277 (10)	0.0170 (3)	
H21	0.708 (2)	0.6374 (13)	0.4558 (15)	0.020*	
C21	0.59630 (17)	0.71486 (10)	0.52676 (12)	0.0171 (3)	
N22	0.65944 (15)	0.69253 (8)	0.62711 (11)	0.0172 (3)	
H22	0.630 (2)	0.7231 (13)	0.6794 (16)	0.021*	
C22	0.77077 (18)	0.63670 (10)	0.65070 (12)	0.0178 (3)	
C23	0.8064 (2)	0.62499 (11)	0.76915 (12)	0.0223 (4)	
H23A	0.7462	0.6636	0.8114	0.027*	
H23B	0.9154	0.6361	0.7847	0.027*	
C24	0.7692 (2)	0.53829 (12)	0.80244 (14)	0.0308 (4)	
H24A	0.6617	0.5264	0.7825	0.037*	
H24B	0.8335	0.5000	0.7627	0.037*	
C25	0.7954 (4)	0.52563 (15)	0.92284 (17)	0.0580 (8)	
H25A	0.7694	0.4697	0.9415	0.070*	
H25B	0.7314	0.5632	0.9623	0.070*	
H25C	0.9024	0.5358	0.9424	0.070*	

### Atomic displacement parameters (Å^2^)

**Table:**

	*U*^11^	*U*^22^	*U*^33^	*U*^12^	*U*^13^	*U*^23^
S1	0.0188 (2)	0.0308 (3)	0.0237 (2)	0.00497 (16)	0.00078 (16)	0.01004 (17)
S2	0.0334 (3)	0.0301 (3)	0.0245 (2)	0.01694 (19)	−0.00509 (18)	−0.00387 (18)
O1	0.0216 (6)	0.0399 (8)	0.0223 (6)	0.0105 (5)	0.0046 (5)	0.0104 (5)
O2	0.0225 (6)	0.0264 (7)	0.0184 (6)	0.0085 (5)	−0.0012 (4)	−0.0018 (5)
C1	0.0168 (7)	0.0135 (8)	0.0153 (7)	0.0021 (6)	0.0025 (6)	0.0011 (6)
C2	0.0181 (8)	0.0137 (8)	0.0144 (7)	0.0028 (6)	−0.0005 (6)	−0.0001 (6)
C3	0.0182 (8)	0.0201 (9)	0.0236 (8)	0.0003 (6)	0.0022 (6)	0.0020 (6)
C4	0.0268 (9)	0.0219 (9)	0.0200 (8)	0.0017 (7)	0.0073 (6)	0.0058 (7)
C5	0.0289 (9)	0.0213 (9)	0.0130 (7)	0.0052 (7)	0.0006 (6)	0.0007 (6)
C6	0.0191 (8)	0.0172 (8)	0.0175 (7)	0.0012 (6)	−0.0026 (6)	−0.0011 (6)
N11	0.0152 (6)	0.0189 (7)	0.0138 (6)	0.0005 (5)	−0.0007 (5)	0.0040 (5)
C11	0.0175 (8)	0.0155 (8)	0.0145 (7)	−0.0034 (6)	−0.0010 (6)	−0.0020 (6)
N12	0.0137 (7)	0.0200 (7)	0.0168 (6)	0.0006 (5)	0.0021 (5)	0.0035 (5)
C12	0.0216 (8)	0.0201 (8)	0.0169 (7)	0.0026 (6)	0.0017 (6)	0.0010 (6)
C13	0.0256 (9)	0.0324 (10)	0.0242 (8)	0.0047 (7)	0.0063 (7)	0.0108 (7)
C14	0.0391 (10)	0.0280 (10)	0.0187 (8)	0.0083 (8)	0.0043 (7)	0.0074 (7)
C15	0.0663 (16)	0.0377 (12)	0.0303 (10)	0.0012 (11)	0.0033 (10)	−0.0059 (9)
N21	0.0153 (7)	0.0195 (7)	0.0159 (6)	0.0045 (5)	−0.0027 (5)	−0.0006 (5)
C21	0.0147 (7)	0.0185 (8)	0.0180 (7)	−0.0001 (6)	0.0001 (6)	0.0006 (6)
N22	0.0190 (7)	0.0177 (7)	0.0148 (6)	0.0015 (5)	−0.0010 (5)	−0.0029 (5)
C22	0.0186 (8)	0.0168 (8)	0.0178 (7)	−0.0021 (6)	−0.0028 (6)	0.0003 (6)
C23	0.0296 (9)	0.0203 (9)	0.0167 (8)	0.0036 (7)	−0.0047 (6)	−0.0010 (6)
C24	0.0442 (11)	0.0265 (10)	0.0215 (8)	−0.0072 (8)	0.0001 (8)	0.0024 (7)
C25	0.115 (2)	0.0344 (13)	0.0240 (10)	−0.0185 (14)	−0.0110 (12)	0.0085 (9)

### Geometric parameters (Å, °)

**Table:**

S1—C11	1.6763 (16)	C13—H13A	0.9900
S2—C21	1.6566 (16)	C13—H13B	0.9900
O1—C12	1.224 (2)	C14—C15	1.516 (3)
O2—C22	1.234 (2)	C14—H14A	0.9900
C1—C6	1.395 (2)	C14—H14B	0.9900
C1—C2	1.401 (2)	C15—H15A	0.9800
C1—N11	1.4207 (19)	C15—H15B	0.9800
C2—C3	1.385 (2)	C15—H15C	0.9800
C2—N21	1.4325 (19)	N21—C21	1.337 (2)
C3—C4	1.393 (2)	N21—H21	0.83 (2)
C3—H3	0.9500	C21—N22	1.395 (2)
C4—C5	1.384 (3)	N22—C22	1.370 (2)
C4—H4	0.9500	N22—H22	0.87 (2)
C5—C6	1.394 (2)	C22—C23	1.503 (2)
C5—H5	0.9500	C23—C24	1.527 (3)
C6—H6	0.9500	C23—H23A	0.9900
N11—C11	1.334 (2)	C23—H23B	0.9900
N11—H11	0.86 (2)	C24—C25	1.516 (3)
C11—N12	1.395 (2)	C24—H24A	0.9900
N12—C12	1.382 (2)	C24—H24B	0.9900
N12—H12	0.84 (2)	C25—H25A	0.9800
C12—C13	1.510 (2)	C25—H25B	0.9800
C13—C14	1.524 (3)	C25—H25C	0.9800
			
C6—C1—C2	119.57 (14)	C15—C14—H14B	108.9
C6—C1—N11	122.61 (14)	C13—C14—H14B	108.9
C2—C1—N11	117.65 (13)	H14A—C14—H14B	107.7
C3—C2—C1	120.47 (14)	C14—C15—H15A	109.5
C3—C2—N21	119.91 (14)	C14—C15—H15B	109.5
C1—C2—N21	119.48 (14)	H15A—C15—H15B	109.5
C2—C3—C4	119.80 (15)	C14—C15—H15C	109.5
C2—C3—H3	120.1	H15A—C15—H15C	109.5
C4—C3—H3	120.1	H15B—C15—H15C	109.5
C5—C4—C3	119.94 (15)	C21—N21—C2	123.85 (14)
C5—C4—H4	120.0	C21—N21—H21	116.0 (13)
C3—C4—H4	120.0	C2—N21—H21	120.1 (13)
C4—C5—C6	120.74 (15)	N21—C21—N22	115.68 (14)
C4—C5—H5	119.6	N21—C21—S2	125.81 (12)
C6—C5—H5	119.6	N22—C21—S2	118.52 (12)
C5—C6—C1	119.45 (15)	C22—N22—C21	128.97 (14)
C5—C6—H6	120.3	C22—N22—H22	117.5 (13)
C1—C6—H6	120.3	C21—N22—H22	113.1 (13)
C11—N11—C1	127.91 (14)	O2—C22—N22	122.64 (14)
C11—N11—H11	114.1 (12)	O2—C22—C23	122.56 (15)
C1—N11—H11	117.4 (13)	N22—C22—C23	114.77 (14)
N11—C11—N12	114.78 (14)	C22—C23—C24	110.15 (14)
N11—C11—S1	127.73 (12)	C22—C23—H23A	109.6
N12—C11—S1	117.49 (11)	C24—C23—H23A	109.6
C12—N12—C11	128.39 (14)	C22—C23—H23B	109.6
C12—N12—H12	115.2 (13)	C24—C23—H23B	109.6
C11—N12—H12	116.3 (13)	H23A—C23—H23B	108.1
O1—C12—N12	122.84 (14)	C25—C24—C23	111.58 (16)
O1—C12—C13	122.44 (15)	C25—C24—H24A	109.3
N12—C12—C13	114.71 (14)	C23—C24—H24A	109.3
C12—C13—C14	112.57 (15)	C25—C24—H24B	109.3
C12—C13—H13A	109.1	C23—C24—H24B	109.3
C14—C13—H13A	109.1	H24A—C24—H24B	108.0
C12—C13—H13B	109.1	C24—C25—H25A	109.5
C14—C13—H13B	109.1	C24—C25—H25B	109.5
H13A—C13—H13B	107.8	H25A—C25—H25B	109.5
C15—C14—C13	113.35 (16)	C24—C25—H25C	109.5
C15—C14—H14A	108.9	H25A—C25—H25C	109.5
C13—C14—H14A	108.9	H25B—C25—H25C	109.5
			
C6—C1—C2—C3	−1.7 (2)	C11—N12—C12—O1	−2.4 (3)
N11—C1—C2—C3	173.81 (15)	C11—N12—C12—C13	178.22 (16)
C6—C1—C2—N21	−177.47 (14)	O1—C12—C13—C14	31.9 (2)
N11—C1—C2—N21	−2.0 (2)	N12—C12—C13—C14	−148.67 (15)
C1—C2—C3—C4	1.9 (2)	C12—C13—C14—C15	67.2 (2)
N21—C2—C3—C4	177.67 (15)	C3—C2—N21—C21	113.70 (18)
C2—C3—C4—C5	−0.8 (3)	C1—C2—N21—C21	−70.5 (2)
C3—C4—C5—C6	−0.4 (3)	C2—N21—C21—N22	173.38 (14)
C4—C5—C6—C1	0.6 (2)	C2—N21—C21—S2	−6.7 (2)
C2—C1—C6—C5	0.4 (2)	N21—C21—N22—C22	6.5 (2)
N11—C1—C6—C5	−174.83 (15)	S2—C21—N22—C22	−173.42 (13)
C6—C1—N11—C11	−49.1 (2)	C21—N22—C22—O2	1.0 (3)
C2—C1—N11—C11	135.57 (16)	C21—N22—C22—C23	−177.18 (15)
C1—N11—C11—N12	−174.00 (14)	O2—C22—C23—C24	−61.4 (2)
C1—N11—C11—S1	5.6 (2)	N22—C22—C23—C24	116.77 (17)
N11—C11—N12—C12	6.0 (2)	C22—C23—C24—C25	−176.8 (2)
S1—C11—N12—C12	−173.65 (13)		

### Hydrogen-bond geometry (Å, °)

**Table:**

*D*—H···*A*	*D*—H	H···*A*	*D*···*A*	*D*—H···*A*
N11—H11···O1	0.86 (2)	1.90 (2)	2.6336 (17)	142.6 (17)
N12—H12···O2^i^	0.84 (2)	2.19 (2)	3.0309 (18)	175.3 (19)
N21—H21···O2	0.83 (2)	1.98 (2)	2.6616 (18)	139.1 (18)
N22—H22···S1^ii^	0.87 (2)	2.75 (2)	3.6147 (14)	172.0 (17)

Symmetry codes: (i) *x*−1, *y*, *z*; (ii) *x*+1/2, −*y*+3/2, *z*+1/2.
